# Virulence gene profiling of enterohemorrhagic (EHEC) and enteropathogenic (EPEC) *Escherichia coli *strains: a basis for molecular risk assessment of typical and atypical EPEC strains

**DOI:** 10.1186/1471-2180-11-142

**Published:** 2011-06-21

**Authors:** Marie Bugarel, Annett Martin, Patrick Fach, Lothar Beutin

**Affiliations:** 1 ANSES (French Agency for Food, Environmental and Occupational Health Safety), Food Safety Laboratory, 23 Av du Général De Gaulle, Fr-94706 Maisons-Alfort, France; 2 Epidemiology, Biostatistics and Mathematical Modelling, Scientific Services, Federal Institute for Risk Assessment (BfR), Diedersdorfer Weg 1 D-12277 Berlin, Germany; 3 National Reference Laboratory for Escherichia coli (NRL-E.coli), Federal Institute for Risk Assessment (BfR), Diedersdorfer Weg 1 D-12277 Berlin, Germany

## Abstract

**Background:**

Enterohaemorrhagic *E. coli *(EHEC) can cause severe disease such as bloody diarrhoea and haemolytic uraemic syndrome in humans. Besides production of Shiga toxins, the presence of LEE (*eae*-gene) and non-LEE (*nle*) encoded effector genes harboured on O-islands OI-122, OI-71 and OI-57 is associated with EHEC virulence and their frequency in outbreaks. Genes encoded by the EHEC-plasmid are putative virulence markers of EHEC. EHEC-plasmids, LEE and non-LEE effector genes have also been detected in some strains of enteropathogenic *E. coli *(EPEC). The objective of this study was to analyze the relationship between EHEC and EPEC for virulence genes encoded by genomic O-islands and by the EHEC-plasmids.

**Results:**

Nle genes *ent/espL2*, *nleB *and *nleE *(OI-122), *nleA*, *nleF *and *nleH1-2 *(OI-71), *nleG5-2 *and *nleG6-2 *(OI-57), *espK *(CP-933N) and the EHEC-plasmid encoded genes *ehxA*, *espP*, *etpD *and *katP *were searched in 73 typical and in 235 atypical enteropathogenic *E. coli *(EPEC) strains. Typical and atypical EPEC each fall into two clusters. Cluster 1 typical (n = 46) and atypical (n = 129) EPEC strains were characterized by the presence of OI-122 encoded genes and grouped together with 64 investigated EHEC strains. Cluster 2 typical (n = 27) and atypical (n = 106) strains grouped together with 52 LEE-negative, Shiga toxin-producing *E. coli *(STEC) and with 21 apathogenic *E. coli *strains. Typical EPEC Cluster 1 strains belonged to serotypes frequently involved in severe illness and outbreaks in children (O111:H2, O114:H2, O55:H6, O127:H6 and O142:H6). Atypical EPEC Cluster 1 strains were characterized by serotypes related to EHEC (O26:H11, O55:H7, O145:H28, O103:H2 and O103:H25).

**Conclusion:**

The OI-122 encoded *nleB *gene was found to be most closely associated with Cluster 1 strains and may serve as a diagnostic tool for the identification of virulent EHEC and EPEC seropathotypes. OI-71 encoded genes *nleA*, *nleF *and *nleH1-2 *are less associated with Cluster 1 strains. EHEC-plasmid, OI-57 and CP-933 associated genes showed only weak similarities with virulent Cluster 1 EHEC and EPEC strains.

## Background

*Escherichia coli *strains that cause diarrhoea in humans have been divided into different pathotypes according to their virulence attributes and the mechanisms involved in the disease process [1,2]. Five major groups of intestinal pathogenic strains have been established, such as enteropathogenic *E. coli *(EPEC), enterohemorrhagic *E. coli *(EHEC), enteroaggregative *E. coli *(EAEC), enterotoxigenic *E. coli *(ETEC) and enteroinvasive *E. coli *(EIEC).

While EPEC is a major cause of infantile diarrhoea in the developing world, EHEC is associated with foodborne outbreaks in the developed world and can cause bloody diarrhoea, haemorrhagic colitis (HC) and the Haemolytic Uraemic Syndrome (HUS) due to the elaboration of Shiga toxin (Stx). More than 400 *E. coli *serotypes that produce Shiga toxins (STEC) have been described [3]. A small number of these have been shown to be implicated in severe disease such as HC and HUS in humans. A classification scheme has been established to group STEC strains into the five seropathotype groups A-E depending on the severity of disease, the incidence of human infections and the frequency of their involvement in outbreaks [4].

Strains belonging to the EPEC group have been subdivided into typical and atypical EPEC as these differ from each other in their adherence mechanisms to human epithelial cells [5] and in their evolutionary lineages [6]. Typical EPEC adhere in a localized manner mediated by bundle-forming pili that are encoded by EAF (EPEC adherence factor) type plasmids harboured by these strains [5,6]. Atypical EPEC do not carry EAF plasmids and most of these adhere in a localized adherence-like pattern to epithelial cells [5]. Some EPEC strains share similarities with certain EHEC strains in terms of their O:H serotypes, virulence genes and other phaenotypical traits [5,7,8].

The chromosomally encoded locus of enterocyte effacement (LEE) which is present in both, EPEC and EHEC strains plays a major role in their pathogenesis. The LEE carries genes for the attaching and effacing phenotype promoting bacterial adhesion and the destruction of human intestinal enterocytes [2,7,9,10]. Besides LEE encoded genes, a large number of non-LEE effector genes have been found on prophages and on integrative elements in the chromosome of the typical EPEC strains B171-8 (O111:NM) [11] and 2348/69 (O127:H6) [12]. In a homology-based search, all non-LEE effector families, except *cif*, found in the typical EPEC strains were also present in EHEC O157:H7 Sakai strain [11,12]. On the other hand, some strain specific effectors were only present in EHEC O157:H7 (EspK, EspX) and not in the EPEC strains. Moreover, EPEC O111 and O127 strains were different from each other regarding the presence of some effector genes (EspJ, EspM, EspO, EspV, EspW, NleD, OspB and EspR) [11,12].

It has been shown that EHEC O157:H7 has evolved stepwise from an atypical EPEC O55:H7 ancestor strain [13,14]. Atypical EPEC and EHEC strains of serotypes O26, O103, O111 and O145 have been found to be similar in virulence plasmid encoded genes, *tir*-genotypes, *tccP *genes, LEE and non-LEE encoded genes indicating that these are evolutionarily linked to each other [8,15-19]. The classification of these strains into the EPEC or the EHEC group is merely based on the absence or presence of genes encoding Shiga toxins (Stx) 1 and/or 2. In EHEC strains, *stx*-genes are typically harboured by transmissible lambdoid bacteriophages and the loss of *stx*-genes has been described to be frequent in the course of human infection with EHEC [20,21]. On the other hand, it has been demonstrated that *stx*-encoding bacteriophages can convert non-toxigenic O157 and other *E. coli *strains into EHEC [22,23].

A molecular risk assessment (MRA) concept has been developed to identify virulent EHEC strains on the basis of non-LEE effector gene typing [24] and a number of *nle *genes such as *nleA*, *nleB*, *nleC*, *nleE*, *nleF*, *nleG2*, *nleG5*, *nleG6*, *nleH1-2 *and *ent/espL2 *have been found to be significantly associated with EHEC strains causing HUS and outbreaks in humans [4,16,17,24].

We recently investigated 207 EHEC, STEC, EPEC and apathogenic *E. coli *strains for the presence of *nle *genes and EHEC virulence plasmid-associated genes. By statistical analysis, two clusters of strains were obtained. OI-122 encoded genes *ent/espL2*, *nleB *and *nleE *were most characteristic for Cluster 1, followed by OI-71 encoded genes *nleH1-2*, *nleA *and *nleF*. EHEC-plasmid encoded genes *katP*, *etpD*, *ehxA*, *espP*, *saa *and *subA *showed only medium to low influence on the formation of clusters. Cluster 1 was formed by all EHEC (n = 44) and by eight of twenty-one EPEC strains investigated, whereas Cluster 2 gathered all LEE-negative STEC (n = 111), apathogenic *E. coli *(n = 30) and the remaining thirteen EPEC strains [17]. These findings indicate that some EPEC strains share non-LEE encoded virulence properties with O157:H7 and other EHEC strains. Such EPEC strains could be derivatives of EHEC which have lost their *stx*-genes but could also serve as a reservoir for the generation of new EHEC strains by uptake of *stx*-phages [16,20,25,26].

To classify strains of the EPEC group according to their relationship to EHEC we have investigated 308 typical and atypical EPEC strains for the presence of *nle*-genes of O-islands OI-57, OI-71 and OI-122, as well as prophage and EHEC-plasmid-associated genes. OI-122 encoded genes were found to be significantly associated with atypical EPEC strains that showed close similarities to EHEC regarding their serotypes and other virulence traits. In typical EPEC, the presence of O-island 122 was significantly associated with strains which are frequently the cause of outbreaks and severe disease in humans.

## Results

### Cluster analysis of EHEC, EPEC, STEC and apathogenic *E. coli *strains

**Figure 1:**
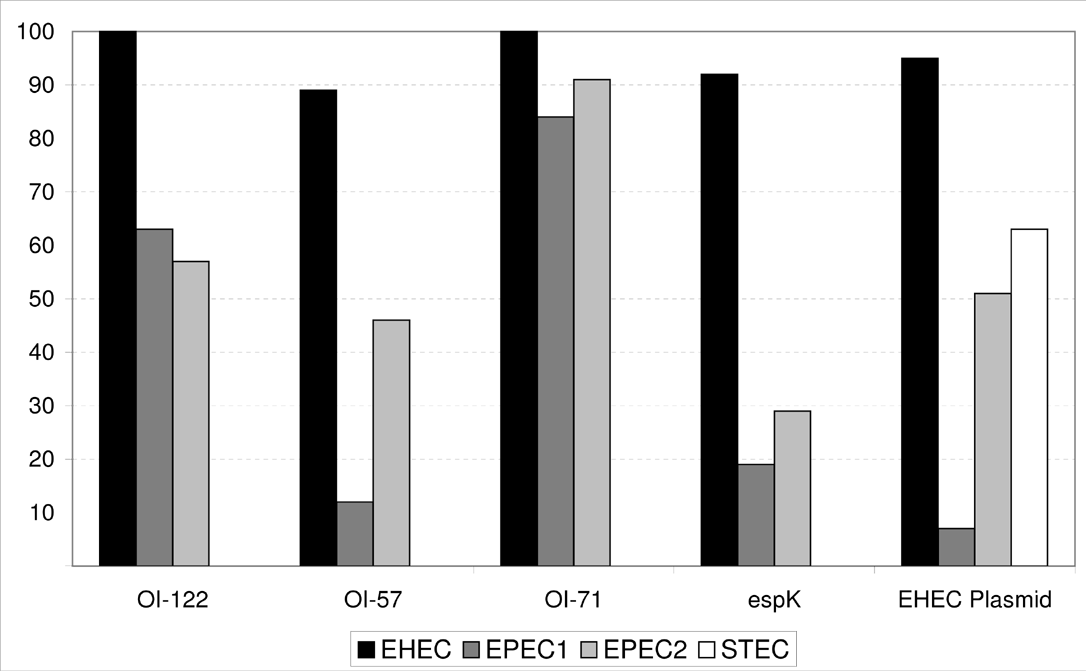
**Distribution of PAIs and the EHEC-plasmid according to *E. coli *pathogroups. **Frequency (% Y-axis) of strains harbouring respective PAIs and the EHEC-plasmid.

*E. coli *pathogroups were established as described in the Methods section. The frequencies and associations between virulence genes and *E. coli *pathogroups are presented in Table 1. The linkage of genes according to their respective PAI or the EHEC-plasmid was 94.7% (230/243) for OI-122, 41.8% (142/340) for OI-71, 46.2% (80/173) for OI-57 and 1.8% (4/220) for the EHEC-plasmid. As not all PAIs were found to be genetically conserved we decided to perform the cluster analysis on single genes. The results from the cluster analysis using thirteen virulence genes that were taken as cluster variables are presented in Table 2. The 445 strains belonging to 151 different serotypes divided into two clusters. Cluster 1 encompassed all 64 EHEC strains, as well as 46 (63%) of the typical and 129 (54.9%) of the atypical EPEC strains. The remaining 133 EPEC strains, as well as all STEC (n = 52) and apathogenic *E. coli *(n = 21) were grouped into Cluster 2. The distribution of PAIs and the EHEC-plasmid according to *E. coli *pathogroups is presented in Figure 1.

**Table 1:** Frequency and associations between virulence genes and *E. coli *pathogroups

Genetic element	Virulence gene	EHEC (n = 64) **n, % (95%-CI)**^**a**^	typical EPEC (n = 73) **n, % (95%-CI)**^**a**^	atypical EPEC (n = 235) **n, % (95%-CI)**^**a**^	STEC (n = 52) **n, % (95%-CI)**^**a**^	*E. coli *(n = 21) **n, % (95%-CI)**^**a**^
pMAR2 [12]	*bfpA*	0, 0 (0;5.6)	68^b ^, 93.2 ^c ^(84.7;97.7)	0, 0 (0;1.6)	0, 0 (0;6.8)	0, 0 (0;16.1)
pO157 [46]	*ehxA*	61, 95.3^c ^(86.9;99.0)	0, 0 (0;4.9)	65, 27.7 (22.0;33.9)	26, 50.0 ^c ^(35.8;64.2)	0, 0 (0;16.1
pO157 [46]	*espP*	37, 57.8^c ^(44.8;70.1)	1, 1.4 (0.03;7.4)	26, 11.1 (7.4;15.8)	14, 26.9^c ^(15.6;41.0)	0, 0 (0;16.1)
pO157 [46]	*etpD*	19, 29.7^c ^(18.9;42.4)	3, 4.1 (0.86;11.5)	79, 33.6^c ^(27.6;40.0)	0, 0 (0;6.8)	0, 0 (0;16.1)
pO157 [46]	*katP*	36, 56.3^c ^(43.3;68.6)	1, 1.4 (0.03;7.4)	40, 17 (12.4;22.4)	1, 1.9 (0.05;10.3)	0, 0 (0;16.1)
OI-71 [31]	*nleA*	47, 73.4^c ^(60.9;83.7)	17, 23.3 (14.2;34.6)	119, 50.6^c ^(44.1;57.2)	0, 0 (0;6.8)	0, 0 (0;16.1)
OI-71 [31]	*nleF*	45, 70.3^c ^(57.6;81.1)	19, 26 (16.5;37.6	87, 37 (30.8;43.5)	0, 0 (0;6.8	0, 0 (0;16.1)
OI-71 [31]	*nleH1-2*	63, 98.4^c ^(91.6;100.0)	60, 82.2 (71.5;90.2)	205, 87.2^c ^(82.3;91.2)	0, 0 (0;6.8)	0, 0 (0;16.1)
OI-122 [31]	*ent/espL2*	64, 100.0^c ^(94.4;100.0)	46, 63^c ^(50.9;74.0)	129, 54.9 (48.3;61.4)	0, 0 (0;6.8)	0, 0 (0;16.1)
OI-122 [31]	*nleB*	64, 100.0^c ^(94.4;100.0)	46, 63^c ^(50.9;74.0)	129, 54.9 (48.3;61.4)	0, 0 (0;6.8)	0, 0 (0;16.1
OI-122 [31]	*nleE*	59, 92.2^c ^(82.7;97.4)	46, 63^c ^(50.9;74.0)	128, 54.5 (47.9;61.0)	0, 0 (0;6.8)	0, 0 (0;16.1)
OI-57 [31]	*nleG5*	33, 51.6^c ^(38.7;64.2)	9, 12.3 (5.8;22.1)	38, 16.2 (11.7;21.5)	0, 0 (0;6.8)	0, 0 (0;16.1)
OI-57 [31]	*nleG6-2*	57, 89.1^c ^(78.7;95.5)	9, 12.3 (5.8;22.1)	107, 45.5^c ^(39.0;52.1)	0, 0 (0;6.8)	0, 0 (0;16.1)
CP-933N [31]	*espK*	59, 92.2^c ^(82.7;97.4)	14, 19.2 (10.9;30.1)	68, 28.9 (23.2;35.2)	0, 0 (0;6.8)	0, 0 (0;16.1)
Stx-phage [47]	*stx*_1_	39, 60.9^c ^(47.9;72.9)	0, 0 (0;4.9)	0, 0 (0;1.6)	18, 34.6^c ^(22.0;49.1)	0, 0 (0;16.1)
Stx-phage [31]	*stx*_2_	33, 51.6^c ^(38.7;64.2)	0, 0 (0;4.9)	0, 0 (0;1.6)	48, 92.3^c ^(81.5;97.9)	0, 0 (0;16.1)
LEE [31]	*eae*	64, 100.0^c ^(94.4;100.0)	73, 100^c ^(95.1;100.0)	235, 100^c ^(98.4;100.0)	0, 0 (0;6.8)	0, 0 (0;16.1)

a) absolute (n) and relative frequencies (%) are shown and the exact 95% confidence level (95%-CI) [48]; b) five strains have lost the EAF plasmid encoding *bfpA *upon subculture; c) standardized residuals > 1 indicates a major influence on a significant chi-square test.

**Table 2:** Summary of cluster analysis with strains belonging to different *E. coli *pathogroups

	Cluster 1		Cluster 2		Total	
**Pathogroup**	**Strains (%)**	**Serotypes (%)**	**Strains (%)**	**Serotypes (%)**	**Strains **	**Serotypes**

EHEC	64 (100.0)	14 (100)	0 (0)	0	64	14
typical EPEC	46 (63.0)	9 (47.4)	27 (37.0)	12 (63.2)	73	19^a^
atypical EPEC	129 (54.9)	40 (50.0)	106 (45.1)	45 (56.25)	235	80^b^
STEC	0 (0)	0	52 (100.0)	20 (100)	52	20
apathogenic *E. coli^c^*	0 (0)	0	21 (100.0)	18 (100)	21	18
all groups	239	63	206	95	445	151

a) two serotypes grouped into Clusters 1 and 2

b) 5 serotypes grouped each in Cluster 1 and Cluster 2.

c) faecal isolates from healthy humans or animals which tested negative for *eae*, *bfpA *and *stx*-genes.

The influence of the different virulence genes on the formation of the "EHEC related" Cluster 1 was calculated using the similarity measure of "Rogers and Tanimoto" [27]. The results are presented in Table 3. The OI-122 encoded genes *nleB*, *ent/espL2 *and *nleE *were highly characteristic of Cluster 1 strains (similarity measure > = 0.947). The OI-71 encoded genes *nleH1-2*, *nleA *and *nleF*, as well as *nleG6-2 *(OI-57) and *espK *(CP-933N) were also found to be characteristic of Cluster 1 strains but to a lesser degree (similarity measure 0.511-0.684). The presence of the EHEC-plasmid pO157 associated genes and of *nleG5-2 *(OI-57) had a minor effect on the formation of Cluster 1 (similarity measure 0.382-0.445).

**Table 3:** Similarity measure between virulence genes and Cluster 1 *E. coli *strains from all groups.

**Genetic element**^**a**^	Virulence gene	**Similarity measure**^**b**^
OI-122	*nleB*	1.000
OI-122	*ent/espL2*	0.991
OI-122	*nleE*	0.947
OI-71	*nleH1-2*	0.684
OI-71	*nleF*	0.621
OI-71	*nleA*	0.553
OI-57	*nleG6-2*	0.527
CP-933N	*espK*	0.511
pO157	*ehxA*	0.445
OI-57	*nleG5-2*	0.440
pO157	*etpD*	0.402
pO157	*espP*	0.399
pO157	*katP*	0.382

a) harbouring the virulence gene; b) A value of 1 indicates complete similarity, while a value of zero means no similarity [49].

### Characteristics of typical EPEC belonging to Clusters 1 and 2

Forty-six (63%) of the 73 typical EPEC strains belonging to nine different serotypes were grouped into Cluster 1. Cluster 2 comprised 27 strains belonging to 12 serotypes (Table 2). Typical EPEC Cluster 1 strains were all positive for OI-122 encoded genes *ent/espL2*, *nleB *and *nleE *(similarity measure 1.0), as well as for *nleH1-2 *(OI-71) (similarity measure 0.678) (Table 4). These genes were absent in typical EPEC Cluster 2 strains, except for *nleH1-2 *(23.3% positive). All other genes that were investigated showed only low similarity (< 0.5) to Cluster 1 (Table 4).

**Table 4:** Similarity measure between virulence genes and Cluster 1 for typical EPEC strains

**Genetic element**^**a**^	Virulence gene	**Similarity measure**^**b**^
OI-122	*ent/espL2*	1.000
OI-122	*nleB*	1.000
OI-122	*nleE*	1.000
OI-71	*nleH1-2*	0.678
OI-71	*nleA*	0.352
OI-71	*nleF*	0.352
OI-57	*nleG5-2*	0.327
OI-57	*nleG6-2*	0.327
CP-933N	*espK*	0.315
pO157	*etpD*	0.259
pO157	*espP*	0.237
pO157	*ehxA*	0.227
pO157	*katP*	0.217

a) harbouring the virulence gene; b) A value of 1 indicates complete similarity, while a value of zero means no similarity [49].

The 73 typical EPEC strains encompassed nineteen different serotypes and one strain was O-rough (Tables 5 and 6). A serotype-specific association with Clusters 1 and 2 was observed. Except for EPEC O119:H6, strains belonging to classical EPEC serotypes such as O55:H6, O111:H2, O114:H2 and O127:H6 grouped in Cluster 1 (Table 5), whereas more rarely observed serotypes were predominant among Cluster 2 strains (Table 6). The single O111:H2 and the O126:H27 strain assigned to Cluster 2 were both negative for all OI-122 associated genes. All other 17 serotypes of typical EPEC were associated with only one cluster each.

**Table 5:** Serotypes of typical EPEC Cluster 1 strains

**Serotype**^**a**^	No. strains	%
**O55:H6**	5	10.9
O66:H8	1	2.2
**O111:[H2]**	17	37.0
O111:H25	2	4.3
O114:H2^b^	11	23.9
O119:H2^c^	4	8.7
**O126:H27**	1	2.2
O127:H6	1	2.2
O142:[H6]	3	6.5
Orough:[H8]	1	2.2
total	46	100.0

a) All strains were isolated from human faeces. In bold: serotypes previously associated with Stx-production [3]. H-type in [brackets] indicates presence of non-motile strains that were investigated for their *fliC *genotype [44].

b) two O114:H2 strains were positive for the EHEC virulence plasmid associated *etpD *gene.

c) EPEC O119:H2 were previously reported as atypical EPEC reacting with *bfpA *probe [5]. One each of the O119:H2 strains was positive for EHEC virulence plasmid associated genes *espP *and *etpD*

**Table 6:** Serotypes of typical EPEC Cluster 2 strains

**Serotype**^**a**^	No. strains	%
O55:[H51]	1	3.7
O86:H8	5	18.5
O86:[H34]	4	14.8
**O111:H2**	1	3.7
O111:[H9]	3	11.1
O118:H5^b^	1	3.7
**O119:[H6]**	4	14.8
O119:[H52]	1	3.7
**O126:H27**	1	3.7
O142:H34	1	3.7
O157:[H45]	4	14.8
O186:[H45]	1	3.7
Total	27	100.0

a) All strains were from human faeces, except the O186:H45 strain which was from faeces of a domestic cat. In bold: serotypes previously associated with Stx-production [3]. H-type in [brackets] indicates presence of non-motile strains that were were investigated for their *fliC *genotype [44].

b) this strain was positive for the EHEC virulence associated *katP *gene

### Characteristics of atypical EPEC belonging to Clusters 1 and 2

A total of 235 atypical EPEC strains were investigated (Table 2). Of these, 129 (54.9%) grouped into Cluster 1. The presence of OI-122 associated genes had the most influence on the formation of atypical EPEC Cluster 1 strains (similarity measures 0.942-1.0, Table 7). By contrast, only four (3.8%) of the 106 atypical EPEC of Cluster 2 were positive for OI-122 genes *ent/espL2 *(one O125:H6 strain) and *nleE *(one Ont:H52, O157:H39 and O168:H33 strain) and none of the strains was positive for *nleB*.

**Table 7:** Similarity measure between virulence genes and Cluster 1 for atypical EPEC strains

**Genetic element**^**a**^	Virulence factor	**Similarity measure**^**b**^
OI-122	*nleB*	1.000
OI-122	*ent/espL2*	0.983
OI-122	*nleE*	0.942
OI-71	*nleF*	0.649
OI-71	*nleA*	0.511
OI-71	*nleH1-2*	0.492
OI-57	*nleG6-2*	0.429
pO157	*ehxA*	0.420
CP-933N	*espK*	0.399
pO157	*etpD*	0.395
pO157	*espP*	0.382
OI-57	*nleG5-2*	0.382
pO157	*katP*	0.313

a) Harbouring the virulence gene; b) A value of 1 indicates complete similarity, while a value of zero means no similarity [49].

The OI-71 encoded genes had only medium influence (similarity measures 0.492-0.649) on the formation of Cluster 1 and OI-57 and EHEC-plasmid encoded genes were of low influence (similarity measures < 0.5). Interestingly, EHEC-plasmid genes *ehxA *(p < 0.001), *etpD *(p < 0.001), *espP *(p < 0.05) and *katP *(p < 0.01) were significantly more frequent in atypical EPEC (51.5% positive) than in typical EPEC (6.9%) strains (data not shown).

The 235 atypical EPEC strains were divided into 80 different serotypes (Table 2). Twenty-five (10.6%) strains were not typable according to their O-antigens (Tables 8 and 9). With the exception of five serotypes (O26:H11, O55:H7, O103:H2, O128:H2 and O145:H28), all others were associated with one cluster only. Strains belonging to classical EHEC types O26:H11, O103:H2, O145:H28 and O157:H7 (n = 30) were predominant in Cluster 1 (23.3%) (Table 8). Only four strains (3.8%) of classical EHEC serotypes grouped into Cluster 2 (Table 9). These were one avian O26:H11 strain (negative for OI-122 and OI-71 genes), one human O103:H2 and two human O145:H28 strains (all negative for OI-122 and OI-71 genes except for *nleH1-2*).

**Table 8:** Serotypes of atypical EPEC Cluster 1 strains

**Serotype**^**a**^	No. strains	**Origin**^**b**^	%
O2:[H40]	3	h (1), a(2)	2.3
O3:[H8]	3	h (3)	2.3
**O15:[H2]**	2	h (2)	1.6
**O26:[H11]^c^**	20	h (9), a (11)	15.5
**O55:H7**	17	h (17)	13.2
**O70:H11^d^**	5	a (5)	3.9
**O76:[H7]^e^**	5	h (5)	3.9
O80:[H2]^d^	3	a (3)	2.3
O86:H11	2	h (2)	1.6
**O100:[H25]**	2	h (1)^d^, a (1)	1.6
**O103:H2^d^**	2	a (2)	1.6
**O103:H25^d^**	4	a (3), f (1)	3.1
**O111:[H11]^d^**	2	h (2)	1.6
O117:[H40]	3	h (3)	2.3
O118:[H8]	3	h (3)	2.3
O119:[H8]	2	h (1), a (1)^d^	1.6
**O119:[H25]^d^**	3	h (2), a (1)	2.3
O127:[H40]	7	h (7)	5.4
**O128:[H2]**	3	h (3)	2.3
**O145:[H28]^d^**	5	h (4), a (1)	3.9
O156:H8	2	h (1), a (1)^d^	1.6
**O157:[H7]^d^**	3	h (3)	2.3
**O177:H11^d^**	2	a (2)	1.6
Ont:[H2]^d^	2	h (2)	1.6
Ont:[H21]	4	h (4)	3.1
Orough:[H40]	2	h (2)	1.6
single^f^	18	see footnote to table	14.0
total	129		100.0

a) in bold: serotypes previously associated with Stx-production [3]

b) Origin and numbers (in brackets) of strains h = human, a = animal, f = food

c) Four O26:H11 strains from humans and seven from animals were positive for the EHEC-plasmid associated *ehxA *gene.

d) All strains were positive for *ehxA*.

e) One strain was positive for *ehxA*.

f) serotypes and strains represented each by one isolate only: O2:H8 (a), O3:H5 (f), O3:H40 (h), O15:H11 (a), O21:H25 (h), O22:[H7] (h), **O45:H7 (a)**, O71:H40 (h), O76:H41 (a),**O84:[H2] (a)**, O109:H25^d ^(a), O117:H25^d ^(h), O121:H- (h), **O121:H19^d ^(h)**, O127:H8 (h), **O128:H8 (h)**, O153:H14 (a), and Orough:[H7] (h)

**Table 9:** Serotypes of atypical EPEC Cluster 2 strains

**Serotype**^**a**^	No. strains	**Origin**^**b**^	%
O28:[H28]^c^	4	h (4)	3.8
**O49:H10**	3	h (1), a (2)^c^	2.8
**O51:H49**	3	h (3)	2.8
**O55:H7**	2	h (2)	1.9
O63:H6	2	h (2)	1.9
O69:H16	2	a (2)	1.9
O108:H9^d^	6	a (6)	5.7
O111:H19	3	h (3)	2.8
O113:H6	2	h (2)	1.9
O114:[H49]	5	h (5)	4.7
O115:[H38]	3	h (3)	2.8
O123:H45	2	h (2)	1.9
O125:H6	3	h (3)	2.8
**O128:[H2]**	10	h (9), a (1)	9.4
**O145:[H28]^d^**	2	h (2)	1.9
O145:[H34]	5	h (5)	4.7
O157:[H16]	4	h (3), f (1)	3.8
O157:H26	2	h (2)	1.9
Ont:[H2]	3	h (1), a (2)	2.8
Ont:H6	2	a (2)	1.9
single^e^	38	see footnote to table	35,8
Total	106		100.0

a)in bold: serotypes previously associated with Stx-production [3].

b)Origin and numbers (in brackets) of strains h = human, a = animal, f = food

c)One strain was positive for the *ehxA *gene

d)All strains were positive for *ehxA*

e)serotypes and strains represented each by one isolate only: O4:H16 (a), **O5:H- (a)**, O5:H11^d ^(a), O8:[H10] (h), O9:H10 (h), **O26:[H11] (a)**, O37:H10 (a), O45:H9 (h), O62:H9^d ^(a), O65:[H25] (h), O69:[H2) (h), O70:H2 (h), O88:H8 (h), O102:[H19] (h),**O103:H2^d ^(h)**, O119:H9 (h), O123:[H25] (h), O127:[H19] (h), O127:H21 (h), O145:H1^d ^(a), O145:H19^d ^(a), **O150:H8 (f)**, O157:H2 (a), O157:H39 (h), O168:[H33] (h), O177:H26 (h), O177:H6 (a), Ont:H7^d ^(a), Ont:H10 (h), Ont:H11 (a), Ont:H14 (h), Ont:[H24] (h), Ont:[H26] (h), Ont:H40 (h), Ont:[H52] (h), Orough:H6 (h), Orough:H9 (h), and Orough:H10 (h).

Forty (50%) of the 80 serotypes encompassing atypical EPEC were associated with strains carrying one or more of the EHEC-plasmid genes *ehxA*, *katP*, *etpD*, *espP*. EHEC-plasmid genes *etpD *(p < 0.01), *ehxA *(p < 0.001) and *espP *(p < 0.001) were significantly more frequent among strains (89/129 = 69%) and serotypes (28/40 = 70%) belonging to Cluster 1 than in strains (32/106 = 30.2%) and serotypes (15/46 = 32.6%) of Cluster 2 (data not shown).

### Presence of virulence genes in STEC and apathogenic *E. coli *strains

The 52 STEC strains investigated in this study belonged to 20 different serotypes (Table 2). Twelve of these (O113:H4, O113:H21, O118:H12, O146:H28, O153:H25, O174:H8, O22:H8, O22:H16, O76:H19, O8:H19, O91:H10 and O91:H21) were previously described from isolates of human origin [3]. Apart from *stx*-genes, 33 (63.5%) of 52 STEC were positive for one or more of EHEC-plasmid associated genes *ehxA*, *espP *and *katP*. None of the STEC was positive for the plasmid *etpD *gene as for all other *nle*-genes investigated in this study (Table 1). The 21 apathogenic *E. coli *strains belonged to 18 different serotypes (Table 2) and were negative for all virulence markers investigated in this study (Table 1).

## Discussion

The concept of molecular risk assessment [24] has been successfully employed for grouping STEC strains into those that are associated with outbreaks and life-threatening disease in humans and those which cause less severe or are not implicated in human disease. The presence of non-LEE effector genes encoded by O-islands OI-122, OI-71 and OI-57 has been shown to be highly associated with EHEC strains that were frequently involved in outbreaks and severe disease in humans [4,16,17,24,28,29]. In a previous work, we were able to associate the presence of OI-122 and OI-71 encoded genes with an "EHEC-Cluster" comprising forty-four EHEC strains as well as eight of twenty-one EPEC strains investigated [17]. This finding indicates that some EPEC strains are more related to EHEC in their virulence patterns, than others.

In order to explore this relationship between EPEC and EHEC more closely, we investigated larger numbers of strains and serotypes of typical and atypical EPEC for thirteen virulence genes associated with EHEC O157 O-islands OI-122, OI-71, OI-57, the EHEC-plasmid and prophage CP-933N. Genes for *nleG5-2 *and *nleG6-2 *were included since OI-57 specific genes were previously found to be associated with classical EHEC and also with some EPEC strains [24,28]. The prophage CP-933 associated *espK *gene was included since its homologues were found in EHEC O157, O26, O103 and O111, in atypical EPEC O55:H7 but not in typical EPEC O127 and O111 strains [11,12,14,30,31].

Our findings indicate that about half of the typical and atypical EPEC strains and serotypes are closely related to EHEC regarding these virulence attributes (Table 2). The presence of OI-122 encoded genes, followed by OI-71 were most significant for the assignment of EPEC to the "EHEC-related" Cluster 1 confirming data from our previous study performed on a different collection of strains [17]. The OI-57 encoded genes *nleG5-2 *and *nleG6-2*, as well as the *espK *gene were not as strongly associated with Cluster 1, as the OI-122 and OI-71 genes. Recently, the OI-57 associated genes *adfO *and *ckf *were reported to be present in 30 (71%) of 42 investigated EPEC strains but a high variability of OI-57 associated orfs in EPEC strains was observed [28]. This could explain the results of our study, where the OI-57 associated *nleG5-2 *gene was found infrequently in all EPEC, whereas the *nleG6-2 *gene was frequent in atypical EPEC (45.5%) but rarely found in typical EPEC (12.3%) (Table 1). Further work is needed to define the genes of OI-57 that are most suitable for the molecular risk assessment of EHEC and EPEC strains.

In our study, EHEC-plasmids were associated with EHEC, STEC and atypical EPEC, but not with typical EPEC strains. EHEC-plasmids are frequently harboured by classical EHEC but also by many LEE-negative STEC strains [32-34]. Correspondingly, EHEC-plasmid encoded genes *ehxA*, *etpD*, *katP *and *espP *had only a small influence on Cluster 1 formation, confirming results of previous studies [16,17]. In this study, EHEC-plasmid genes were significantly more associated with atypical EPEC Cluster 1 than with Cluster 2 strains. The high proportion of EHEC-plasmid positives among Cluster 1 strains suggests that many of these may have derived from EHEC by losing *stx*-genes. A loss of *stx*-genes was reported to occur frequently in classical EHEC strains [23,26]. EHEC-plasmid genes were found in 23/29 (79.3%) of atypical EPEC Cluster 1 strains belonging to EHEC related serotypes O26:H11, O103:H2, O145:H28 and O157:H7 (data not shown). These 30 EHEC-like strains showed the same virulence characteristics (presence of OI-122 genes) as their homologous EHEC strains.

In addition to this, there are epidemiological findings pointing to a closer relationship between "Cluster 1" atypical EPEC and EHEC strains. Significantly (p < 0.05) more typable (78/120 = 65.0%) Cluster 1 strains than Cluster 2 strains belonged to serotypes (18/40 = 45.0%) that are associated with the production of Shiga toxins (38). Only 26.6% (24/90) of the atypical EPEC strains of Cluster 2 showed O:H types (10/46 = 21.7) previously associated with Stx-production.

Typical EPEC were also found to split into Cluster 1 and Cluster 2 strains. Cluster 1 was formed by typical EPEC serotypes O55:H6, O114:H2, O111:[H2], O127:H6 and O142:H6 strains which accounted worldwide for large outbreaks in hospitals, infant wards and day nurseries with a high mortality rate [35-37]. Cluster 2 typical EPEC accounted for serotypes that were more rarely associated with outbreaks, except for EPEC O119:H6, the latter was frequently associated with infantile diarrhoea in Brazil [38,39]. On the basis of these findings, a seropathotype classification for typical EPEC similar to those described for STEC [4,24] can be established. Typical EPEC strains associated with outbreaks and high mortality are gathered in Cluster 1 which is mainly characterized by the presence of OI-122 associated genes *ent/espL2*, *nleB*, *nleE*. These findings are supported by two clinical studies showing that the presence of OI-122 encoded genes was significantly associated with diarrhoea in patients infected with atypical EPEC [40,41]. The function of *nle*-genes in pathogenesis of EHEC and EPEC infection is only partially known [30,42,43]. Further work is needed to explore the contribution of OI-122 effectors to the high infectivity and virulence of EPEC and EHEC strains resulting in outbreaks and severe disease in humans.

It has been shown previously that the evolution of typical and atypical EPEC has occurred from LEE positive ancestor strains and divergent phylogenetic groups of EPEC (EPEC1 to EPEC4) and EHEC (EHEC1 and EHEC2) were established [1,6,37]. Virulence genes harboured by EAF-plasmids, EHEC-plasmids and *stx*-phages were found in phylogenetically unrelated strains indicating that these were acquired several times during evolution [1]. Their horizontal spread to unrelated strains and the frequent loss of plasmid and bacteriophage inherited determinants makes these less suitable for identifying clones associated with high infectivity and virulence in humans. The OI-122 inherited *nle*-genes were found to be significantly associated with highly virulent Cluster 1 strains of EHEC and EPEC. They appear to be more stably inherited than plasmid and phage associated genes and could thus serve as an additional diagnostic tool for the reliable identification of EHEC and EPEC infections in humans, animals and EHEC contamination of food sources and the environment.

## Conclusion

Our results indicate that the OI-122 pathogenicity island is a common attribute that is significantly associated with highly virulent EHEC and EPEC strains. Of the OI-122 encoded genes, *nleB *was found as most conserved and thus presents a suitable marker for genetic screening for human virulent EHEC and EPEC strains. Horizontally transferred genetic elements such as the virulence-plasmids and phages were less significantly associated with the highly virulent clones of EHEC and EPEC strains.

## Methods

### Bacteria

A total of 445 *E. coli *strains from the collection of the National Reference Laboratory for *Escherichia coli *(NRL-E.coli) were investigated. These originated from humans (n = 286), domestic animals (n = 84) and food (n = 70). Five strains were of unknown origin. The 445 strains were grouped into apathogenic *E. coli *(n = 21), atypical EPEC (n = 235), typical EPEC (n = 73), EHEC (n = 64) and STEC (n = 52) according to the presence or absence of genes encoding Stx (*stx*_1 _+ *stx*_2_), intimin (*eae*) and bundle forming pili (*bfpA*). All strains were investigated for their O (lipopolysaccharide) and H (flagellar) serotypes. Non-motile strains were examined for their flagellar (*fliC*) genotype as previously described [44]. Highly purified total DNA of the strains was prepared from 0.5 ml overnight cultures of bacteria using the RTP^® ^Bacteria DNA Mini Kit (Invitek, Berlin, Germany).

### Detection of genes by real-time PCR

To investigate the presence of seventeen genes previously described as virulence markers of STEC, EPEC and EHEC the real-time PCR method was employed using the GeneDisc^® ^array as previously described [17], or the Applied Biosystems 7500 real time PCR system. Standard cycling conditions (15 sec 94°C, 1 min 60°C and 40 cycles) were used for the Applied Biosystems 7500 system. The primers and probes for the detection of following genes (*stx*_1_, *stx*_2_, *eae*, *ehxA*, *espP etpD*, *katP*, *nleA*, *nleF*, *nleH1-2 ent/espL2*, *nleB*, *nleE*) have been described previously [16]. Primers and probes for the detection of *bfpA*, *nleG5-2*, *nleG6-2 *and *espK *were developed for this work (Table 10). The reference strains for STEC and EHEC were used as previously described [16]. Strain E2348/69 (O127:H6) [12] served as control for typical EPEC and strain CB9615 (O55:H7) [14] as a control of atypical EPEC. *E. coli *K-12 strain MG1655 [45] served as a negative control for the eighteen virulence markers investigated in this work.

**Table 10:** Primers and probes for real-time PCR detection of virulence genes developed for this study

**Target gene**^**a**^	Forward primer, reverse primer and probe sequences (5'-3')	Location within sequences	**Gene Bank accession no**.
*nleG6-2 *(Z2150)	ATATGCTCTCTATATGATAAGGATG	1928877-1928901	AE005174
	AAAGTGACATTCGTCTTTTCTCATA	1928996-1928872	
	[6FAM]CGTTAGTGCAACTTGTTGAAACTGGTGGAA[BHQ1]	1928902-1928931	
*nleG5-2 *(Z2151)	AGACTATTCGTGGAGAAGCTCAAG	1929199-1929222	AE005174
	TATTGAAGGCCAATCTGGATG	1929337-1929317	
	[6FAM]TGGATATTTTATGGGAAGTCTTAACCAGGATGG[BHQ1]	1929269-1929301	
*espK*	ATTGTAACTGATGTTATTTCGTTTGG	1673295-1673320	AE005174
	GRCATCAAAAGCGAAATCACACC	1673419-1673397	
	[6FAM]CAGATACTCAATATCACAATCTTTGATATATAAACGACC[BHQ1]	1673330-1673368	
*bfpA*	CCAGTCTGCGTCTGATTCCA	2756-2775	FM180569
	CGTTGCGCTCATTACTTCTGAA	2816-2795	
	TAAGTCGCAGAATGC-MGB	2777-2791	

a) Z2150 and Z2151 derive from OI-57 [24]

### Definition of *E. coli *pathogroups

The genes *eae*, *stx*_1 _*stx*_2 _and *bfpA *were used to define *E. coli *pathogroups and were therefore not taken as independent variables for the cluster/statistical analysis. On the genotype basis, the strains were grouped as atypical EPEC (*eae *only), typical EPEC (*eae *and *bfpA*), STEC (*stx*_1 _and/or *stx*_2_), EHEC (*eae *and *stx*_1 _and/or *stx*_2_) and apathogenic *E. coli *(absence of *eae*, *bfpA*, *stx*_1 _and *stx*_2_). The presence of the following genes was used to define clusters of EHEC related and EHEC unrelated strains as previously described [17]: *ehxA*, *espP*, *etpD *and *katP *(EHEC-virulence plasmid pO157), *nleA*, *nleF *and *nleH1-2 *(OI-71), *ent/espL2*, *nleB *and *nleE *(OI-122). These variables proved to be useful for the characterization of STEC and EHEC strains [4,16,17,24,29]. In addition to this, genes *nleG5-2 *and *nleG6-2 *(OI-57) [24] and *espK *(prophage CP-933N) [31] had previously been found to be associated with EHEC [11,12,24,25,28] and therefore included as new variables for the cluster analysis.

### Statistical analysis

The seventeen virulence genes that were investigated in the 445 *E. coli *strains are listed in Table 1. To analyse the relationship between the seventeen virulence factors investigated in this work and the *E. coli *pathogroups, the presence of the virulence factors was calculated per pathogroup (Table 1). For the analysis of associations between the virulence factors and the *E. coli *pathogroups univariate analysis with a chi-square test was used. If frequencies were low Fisher's exact tests was used for the calculation. As a significance level, α was set to 0.05. All p-values ≤ α were considered statistically significant. To determine which virulence genes were major contributors in the elimination of the null hypothesis we calculated standardized residuals. When the absolute value of the residual is greater than 1.00 we can conclude that there is a major influence on a significant chi-square test between a given pathotype and the respective virulence gene (Table 1). A cluster analysis was performed in order to analyse similarities between the *E. coli *pathogroups. Since the presence or absence of virulence genes is binary scaled, the similarity was calculated according to "Rogers and Tanimoto" [27]. The linkage between groups was selected as the cluster method.

## Competing interests

The authors declare that they have no competing interests.

## Authors' contributions

LB and PF played an integral role in the project conception and MB, PF and LB in method development. MB was mainly responsible for the design and execution of the experimental procedures. Data processing and statistical analysis was done by AM. Data analysis and interpretation of the results was completed by all authors. LB was mostly responsible for the preparation of the manuscript. All authors have read and approved the final manuscript.
